# Comparative supragenomic analyses among the pathogens *Staphylococcus aureus, Streptococcus pneumoniae*, and *Haemophilus influenzae *Using a modification of the finite supragenome model

**DOI:** 10.1186/1471-2164-12-187

**Published:** 2011-04-13

**Authors:** Robert Boissy, Azad Ahmed, Benjamin Janto, Josh Earl, Barry G Hall, Justin S Hogg, Gordon D Pusch, Luisa N Hiller, Evan Powell, Jay Hayes, Susan Yu, Sandeep Kathju, Paul Stoodley, J Christopher Post, Garth D Ehrlich, Fen Z Hu

**Affiliations:** 1 Center for Genomic Sciences, Allegheny-Singer Research Institute, 320 East North Ave, Pittsburgh, PA 15212, USA; 2 Bellingham Research Institute, 218 Chuckanut Point Rd, Bellingham, WA 98229, USA; 3 Joint Carnegie Mellon University - University of Pittsburgh Doctoral Program in Computational Biology, University of Pittsburgh, Pittsburgh, PA, 15260, USA; 4 Fellowship for Interpretation of Genomes, Burr Ridge, IL 60527, USA; 5 Computation Institute, University of Chicago, Chicago, IL 60637, USA; 6 Department of Microbiology and Immunology, Drexel University College of Medicine, Allegheny Campus, Allegheny General Hospital, Pittsburgh, PA 15212, USA; 7 Department of Otolaryngology-Head and Neck Surgery, Drexel University College of Medicine, Allegheny Campus, Allegheny General Hospital, Pittsburgh, PA 15212, USA; 8 Department of Internal Medicine, University of Nebraska Medical Center, Omaha, NE 68198, USA

## Abstract

**Background:**

*Staphylococcus aureus *is associated with a spectrum of symbiotic relationships with its human host from carriage to sepsis and is frequently associated with nosocomial and community-acquired infections, thus the differential gene content among strains is of interest.

**Results:**

We sequenced three clinical strains and combined these data with 13 publically available human isolates and one bovine strain for comparative genomic analyses. All genomes were annotated using RAST, and then their gene similarities and differences were delineated. Gene clustering yielded 3,155 orthologous gene clusters, of which 2,266 were core, 755 were distributed, and 134 were unique. Individual genomes contained between 2,524 and 2,648 genes. Gene-content comparisons among all possible *S. aureus *strain pairs (n = 136) revealed a mean difference of 296 genes and a maximum difference of 476 genes. We developed a revised version of our finite supragenome model to estimate the size of the *S. aureus *supragenome (3,221 genes, with 2,245 core genes), and compared it with those of *Haemophilus influenzae *and *Streptococcus pneumoniae*. There was excellent agreement between RAST's annotations and our CDS clustering procedure providing for high fidelity metabolomic subsystem analyses to extend our comparative genomic characterization of these strains.

**Conclusions:**

Using a multi-species comparative supragenomic analysis enabled by an improved version of our finite supragenome model we provide data and an interpretation explaining the relatively larger core genome of *S. aureus *compared to other opportunistic nasopharyngeal pathogens. In addition, we provide independent validation for the efficiency and effectiveness of our orthologous gene clustering algorithm.

## Background

Most strains of the Gram-positive bacterium *Staphylococcus aureus *are avirulent, antibiotic-sensitive commensals; however, over the past several decades there have emerged a number of pandemic, virulent, antibiotic-resistant strains including methicillin-resistant (MRSA) and vancomycin-resistant (VRSA) strains [1]. Although many *S. aureus *infections originate in the community, it is also the most common nosocomial bacterial infection in U.S. healthcare institutions, accounting for more than half a million hospital-acquired infections annually which exact an enormous financial and healthcare burden.

*S. aureus *can be detected in its primary reservoir in the anterior nares on a regular basis in about 20% (and intermittently in another 60%) of the human population [2], leading to efforts for decolonization in healthcare settings [3]. Some *S. aureus *strains have acquired any of a large number of virulence factors, and can cause a range of infections from mild to serious including pimples, impetigo, boils, cellulitis, endocarditis, necrotizing fasciitis, osteomyelitis, pneumonia, septic arthritis, septicemia, and toxic shock syndrome [4,5]. The widespread, long-term exposure of humans to *S. aureus *antigens from non-pathogenic strains may help explain why the development of an effective vaccine against pathogenic strains of *S. aureus *is difficult [6]. As a result of its ubiquity and its ability to acquire virulence and antibiotic-resistance factors it is now estimated that invasive MRSA infections cause more deaths in the U.S. (18,650 vs. 17,011 in 2005) than HIV/AIDS [7-10]. *S. aureus *infections of domestic livestock are also of concern, and cause significant economic losses [11,12].

Our laboratory has developed the *Distributed Genome Hypothesis *(DGH), a model for understanding intra-species gene content differences in bacterial pathogens, especially those associated with chronic infections [13-15]. The DGH states that pathogenic bacterial species use horizontal gene transfer to make available to the genomes of individual strains a set of non-core *distributed *genes with varying population frequencies, and with varying probabilities of contributing to the species' population fitness. The observation that these distributed genes are present at significantly different frequencies in the population of a given pathogenic species, combined with the fact that the total number of genes available in the population is larger (often much larger) than the number of genes in any one strain's genome, has led us to describe the set of genes available to a bacterial pathogenic species as a *supragenome *[16-18] in preference to the synonymously used term *pan-genome *[19]. The DGH views the combinatorial process of augmenting a set of core genes with a significant number of non-core distributed genes in each strain's genome as an evolutionary strategy to maximize the species' population fitness across a range of environmental conditions (e.g., nutrient supply, competing microbial flora, host innate and adaptive immune responses, and exposure to antibiotics) and at rates that are significantly greater than can be achieved through the vertical transmission and exchange of alleles of a relatively fixed set of genes [20].

Whole genome shotgun sequencing using 454 Life Sciences' next-generation pyrosequencing technology has been used in our laboratory to obtain high-coverage draft genomic DNA sequence datasets for large numbers of strains of several human bacterial pathogens [21-23]. Using these data, a predictive finite supragenome model of the DGH was developed, and has been used to delineate the supragenomes of *Haemophilus influenzae *[21] and *Streptococcus pneumoniae *[22], two species that are naturally transformable. Here we extend our research on the DGH in several respects: (i) by comparatively examining all of the genomes that were available for *S. aureus*, a non-transformable species; (ii) by making use of a newly available automated bacterial genome annotation service (the RAST system) for the annotation of these 17 genomes, a service that is based on a set of well-curated biological subsystem annotations [24]; and (iii) by introducing a revised finite supragenome model that allows the estimates of the population gene frequencies to vary during the maximization of the log-likelihood of the observed sample gene frequency data [21]. We use the descriptive and predictive capabilities of our revised finite supragenome model to delineate the *S. aureus *supragenome, compare it with the supragenomes of *Haemophilus influenzae *and *Streptococcus pneumoniae*, and estimate the number of chromosomal genes that would be found with the sequencing of additional *S. aureus *genomes.

## Results

### Bacterial genomic DNA sequences and the annotation data for their protein-coding genes

Table 1 lists general information about the genomes of the 17 *S. aureus *strains that were used for this supragenome analysis. The following points should be noted about these DNA sequence datasets. First, only the bacterial chromosome DNA sequences from the 14 published *S. aureus *strains were used; the plasmid DNA sequences available for 8 of these 14 strains were not included in the analyses. Second, the 14 published *S. aureus *strains examined included some genomes that are known to be very similar (e.g., strains JH1 and JH9). Third, 16/17 of the *S. aureus *strains whose genomes were examined were of human origin with only one being of livestock origin (RF122, isolated from a case of bovine mastitis).

**Table 1:** Bacterial Chromosome Sequence Datasets Used for Supragenome Analysis

Genome*	Reference	Sensitivity	MBp	Contigs	%GC	Plasmids	Source
CGSSa00	this publication	untested	2.78	18	32.7	unknown	
CGSSa01	this publication	untested	2.86	58	32.6	unknown	elbow arthroplasty infection
CGSSa03	this publication	untested	2.83	68	32.8	unknown	abdominoplasty infection
COL	Gill et al., 2005	MRSA	2.81	1	32.8	1	
JH1	Mwangi et al., 2007	VISA	2.91	1	33.0	1	patient on vancomycin
JH9	Mwangi et al., 2007	VISA	2.91	1	33.0	1	patient on vancomycin
MRSA252	Holden et al., 2004	MRSA	2.90	1	32.8	0	
MSSA476	Holden et al., 2004	MSSA	2.80	1	32.8	1	
Mu3	Neoh et al., 2008	hetero-VISA	2.88	1	32.9	0	
Mu50	Kuroda et al., 2001	HA-MRVISA	2.88	1	32.9	1	pus, neonatal surgical infection
MW2	Baba et al., 2002	CA-MRSA	2.82	1	32.8	0	
N315	Kuroda et al., 2001	MRSA	2.81	1	32.8	1	pharyngeal smear
NCTC8325	Gillaspy et al., 2006	MRSA	2.82	1	32.9	0	
Newman	Baba et al., 2008	MSSA	2.88	1	32.9	0	
RF122 (ET3-1)	Herron-Olson et al., 2007	sensitive	2.74	1	32.8	0	mastitis (bovine)
USA300 (FPR3757)	Diep et al., 2006	CA-MRSA	2.87	1	32.8	3	abscess, HIV + i.v. drug user
USA300TCH15	Highlander et al., 2007	CA-MRSA	2.87	1	32.8	1	asymptomatic pediatric patient

*The NCBI's "genus species [subspecies]" name for each strain is either *Staphylococcus aureus *(for the bovine isolate RF122) or *Staphylococcus aureus subsp. aureus*. **Abbreviations**: Antibiotic sensitivity: CA, community-acquired; HA, healthcare-acquired; M, methicillin; R, resistant; S, sensitive; V, vancomycin; VI, V-intermediate; hetero-VI, heterogeneous VI; SA, *Staphylococcus aureus*.

Automated bacterial genome annotation is the only practical method to keep pace with the productivity of modern DNA sequencing technologies [25] such as those used in this study to obtain high-coverage (~20X) draft genomic sequences for clinical *S. aureus *strains (Table 1). We chose the RAST automated bacterial genome annotation system [24] because it is free of charge, confidential, secure, handles compute-intensive bacterial genome annotation jobs quickly, and allows users to upload their own sequences, i.e., it does not just offer annotations of publicly available bacterial genomic DNA sequence datasets. Currently, as Table 2 shows, even based on the rudimentary criterion of CDS counts, different automated bacterial genome annotation providers can produce significantly different results from the same genomic DNA sequence datasets (and it should be noted that *exact *agreement in Table 2 for CDS counts for a given genome typically indicates re-use of an NCBI RefSeq dataset by another annotation provider). Thus, to have a consistent set of CDS annotations, we used the RAST system to annotate both our in-house generated draft genomic sequences and the bacterial chromosomes of the 14 published *S. aureus *strains. (Table 1).

**Table 2:** Chromosomal Coding Sequence (CDS) Counts From Different Annotation Providers

Genome	PGAAP	RAST	RefSeq		GenBank		CMR-P	CMR-T	IMG
							
			CDS	Accession	CDS	Accession			
CGSSa00	2,781	2,733	n.a	n.a.	n.a	ABWS00000000	n.a	n.a	n.a
CGSSa01	2,971	2,769	n.a	n.a.	n.a	ABWT00000000	n.a	n.a	n.a
CGSSa03	2,951	2,795	n.a	n.a.	n.a	ABWY00000000	n.a	n.a	n.a
COL	2,864	2,687	2,615	NC_002951.2	2,673	CP000046.1	2,712	n.a	2,649
JH1	2,992	2,828	2,747	NC_009632.1	2,747	CP000736.1	n.a	n.a	2,789
JH9	2,997	2,828	2,697	NC_009487.1	2,697	CP000703.1	n.a	n.a	2,731
MRSA252	2,901	2,823	2,656	NC_002952.2	2,744	BX571856.1	2,744	2,689	2,733
MSSA476	2,829	2,679	2,579	NC_002953.3	2,619	BX571857.1	2,619	2,524	2,614
Mu3	2,945	2,777	2,698	NC_009782.1	2,699	AP009324.1	n.a	n.a	2,698
Mu50	2,949	2,785	2,697	NC_002758.2	2,699	BA000017.4	2,714	2,628	2,697
MW2	2,860	2,695	2,632	NC_003923.1	2,632	BA000033.2	2,632	2,849	2,632
N315	2,837	2,688	2,588	NC_002745.2	2,593	BA000018.3	2,592	2,762	2,588
NCTC8325	2,924	2,747	2,892	NC_007795.1	2,892	CP000253.1	2,892	2,654	2,894
Newman	3,025	2,813	2,614	NC_009641.1	2,614	AP009351.1	n.a	n.a	2,614
RF122	2,795	2,715	2,509	NC_007622.1	2,589	AJ938182.1	2,589	2,595	2,579
USA300	2,957	2,778	2,560	NC_007793.1	2,560	CP000255.1	2,578	n.a	2,646
USA300TCH15	2,955	2,783	2,657	NC_010079.1	2,657	CP000730.1	n.a	n.a	2,710

**Abbreviations**: PGAAP, NCBI's "Prokaryotic Genome Automated Annotation Pipeline"; RAST, Argonne National Laboratory's "Rapid Annotation using Subsystem Technology" system; CMR, J. Craig Venter Institute's Comprehensive Microbial Resource (v 21.0); CMR-P and CMR-T, primary annotations and JCVI's re-annotations; IMG, DOE-Joint Genome Institute's Integrated Microbial Genomes (v. 2.5); n.a., not available. A RefSeq is derived from an underlying GenBank record, but the annotations in each record may differ.

### Analyses of *S. aureus *gene frequencies using 17 genomes

We used our previously described computational pipeline [21] to cluster the chromosomal genes from the 17 *S. aureus *genomes which has proven to be highly reliable in comparison with other systems (Donati et al 2010, *vide infra*)[26]. This single-linkage clustering procedure is designed to accommodate the use of draft genomic DNA sequence data and its annotations, i.e., data that may include open reading frames that are disrupted by genome assembly errors, or genes interrupted by contig breaks. This procedure yields clusters of CDS orthologs based on 70% sequence identity over 70% of the shorter sequence. Based on these clustering results, genes were classified (Table 3) based on their frequency as either unique (observed in one genome only), distributed (observed in more than one but not all genomes), or core (present in all genomes).

**Table 3:** Orthologous Clusters and Coding Sequences (CDS) in the *S. aureus *Supragenome

Genome	Orthologous Clusters (genes)				CDS				
		
	All	Distributed	Unique	Non-core %	All	Core	Distributed	Unique	Non-core %
CGSSa00	2,534	266	2	11	2,701	2,410	289	2	11
CGSSa01	2,648	364	18	14	2,733	2,362	353	18	14
CGSSa03	2,628	358	4	14	2,765	2,389	372	4	14
COL	2,543	270	7	11	2,649	2,374	268	7	10
JH1	2,643	377	0	14	2,796	2,382	414	0	15
JH9	2,643	377	0	14	2,796	2,382	414	0	15
MRSA252	2,645	376	3	14	2,788	2,393	392	3	14
MSSA476	2,553	275	12	11	2,643	2,370	261	12	10
Mu3	2,629	363	0	14	2,747	2,369	378	0	14
Mu50	2,629	363	0	14	2,754	2,370	384	0	14
MW2	2,574	302	6	12	2,661	2,370	285	6	11
N315	2,538	271	1	11	2,660	2,362	297	1	11
NCTC8325	2,589	315	8	12	2,712	2,380	323	9	12
Newman	2,579	293	20	12	2,775	2,391	361	23	14
RF122	2,524	205	53	10	2,682	2,391	238	53	11
USA300	2,620	354	0	14	2,744	2,387	357	0	13
USA300TCH15	2,620	354	0	14	2,746	2,390	356	0	13
									
**All 17 strains**	**3,155**	**755**	**134**	**28**	**46,352**	**40,472**	**5,742**	**138**	**13**

Core clusters (2,266 here) have one or more representative CDS in each genome examined; unique clusters are represented in only one genome; and distributed clusters in more than one but not all genomes examined.

The clustering results yielded 3,155 orthologous gene clusters (genes), of which 2,266 were core, 755 were distributed and 134 were unique. The unique genes had an uneven distribution, and it was not surprising that the bovine isolate, RF122, had both the largest number of unique genes (n = 53) and the smallest number of distributed genes (n = 205). Individual genomes contained between 2,524 (RF122) and 2,648 (CGSSa01) genes, whilst the maximum difference between any pair of *S. aureus *genomes, out of all possible (17 choose 2 = 136) pairs, based on protein-encoding gene content, was 476 genes. In addition, although only 13% of the total number of gene annotations among the 17 strains are non-core, 28% of the total number of genes found in these 17 strains are non-core indicating that many of these genes are found repeatedly throughout the species

**Figure 1:**
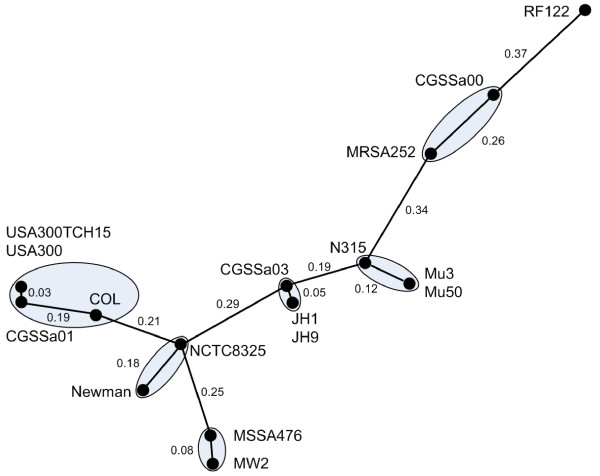
**Clustering of strains using neighbor-grouping analysis**. The figure shows the relationships among the 17 *Staphylococcus aureus *genomes under study based on the percentage of shared distributed gene. Valid *neighbor groups *of genomes (see Materials and Methods) are enclosed in ellipses.

Figure 1 shows the results of a *neighbor grouping *analysis [23] performed using the distributed *S. aureus *genes, a procedure that displays the relatedness of strains based on a metric of identity by state (as opposed to identity by descent) which overcomes the problems associated with trying to do phylogenetic analyses on mosaic genomes resulting from horizontal gene transfer. The edge weights shown in the graph represent the fraction of the distributed genes in the supragenome that is either present in both (or absent in both) of the genomes represented by the vertices of the relevant edge. The mean distance among all possible strains pairs (n = 136) is 0.34 ± 0.01, and valid neighbor *groups *are indicated (see Materials and Methods). This analysis of distributed gene content helped determine the relationship of the three genomes that we sequenced with the 14 published *S. aureus *genomes. The genome of the strain CGSSa01 was very closely related to the genomes of the community-acquired methicillin-resistant *S. aureus *(CA-MRSA) strains USA300 and USA300TCH15, and the genome of the strain CGSSa03 was very closely related to the genomes of the vancomycin-intermediate *S. aureus *(VISA) strains JH1 and JH9.

**Figure 2:**
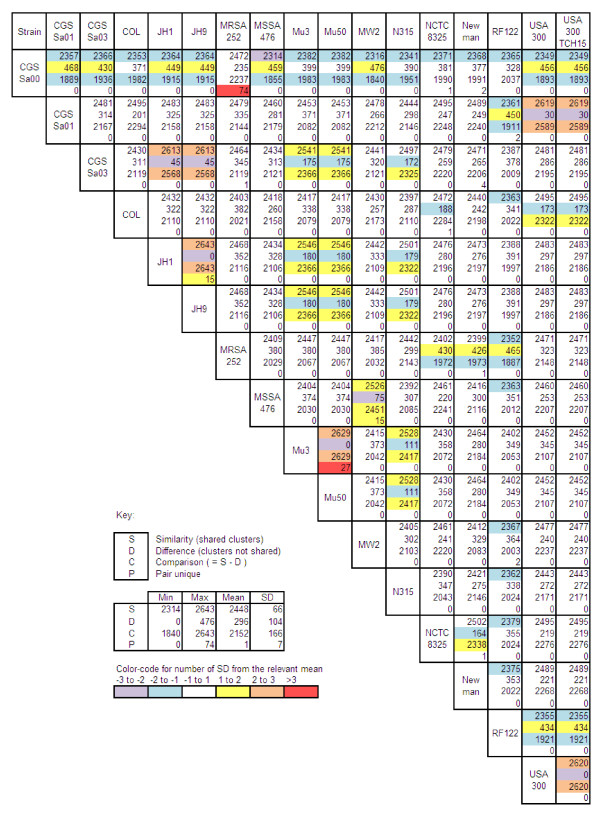
**Pair-wise gene possession comparisons among all 136 possible *Staphylococcus aureus *strains pairs**. The comparison of two strains is summarized in the (4-level) box at the intersection of the row and column corresponding to the respective strains. Pair-wise relationships are summarized based on the number of genes with orthologs in each of the two strains (S = *similarity *score, level 1 of each box); the number of genes with an ortholog in one strain but not the other (D = *difference *score, level 2 of each box); a composite comparison score (C = S - D, level 3 of each box); and the number of genes with orthologs found only in both strains (P = *pair unique *score, level 4 of each box).

Figure 2 summarizes the pair-wise relationships between the genomes. The metrics used to describe these relationships are: (i) the number of genes with orthologs in each of the two strains (S = *similarity *score); (ii) the number of genes with an ortholog in one strain but not the other (D = *difference *score); (iii) a composite *comparison *score (C = S - D); and (iv) the number of genes with orthologs found only in both strains (P = *pair unique *score). The mean similarity and difference scores calculated for all possible pairs (n = 136) produced values of 2,448 and 296 genes, respectively. These results and the occurrence of unique genes (Table 3) are consistent with the known similarities between the JH1 and JH9 genomes [27]; the Mu3 and Mu50 genomes [28]; and the USA300 and USA300TCH15 genomes [29]. These results are also consistent with the expected outlier status of RF122 with respect to the strains of human origin. One interesting pair-wise relationship is that of MRSA252 and CGSSa00; the comparison score was at its maximum for MRSA252, whereas all other values for this score involving CGSSa00 were less than the mean, and in most cases more than one standard deviation below the mean. In addition, the maximum pair unique score for all pair-wise relationships was that observed between CGSSa00 and MRSA252, suggesting a highly significant degree of relatedness.

### Analyses of *S. aureus *population gene frequencies using a revised finite supragenome model

To more accurately model the number of genes contained within a species' supragenome we have revised our finite supragenome model to take advantage of the observed gene frequencies obtained from the sequencing of limited numbers of target strains. Our model treats each of the *N *genes in a bacterial species' supragenome as an independent Bernoulli random variable, with a gene's occurrence in a genome of one of the strains of the species representing a success outcome of a Bernoulli trial that has a probability equal to the gene's population frequency among all strains [21]. We model the population gene frequencies for a species with a supragenome as being limited to *K *classes, with each class defined by two parameters: a Bernoulli probability *μ_k _*that represents the gene frequency, and a corresponding mixture coefficient *π_k _*that represents the probability that one of the *N *genes in the supragenome belongs to class *k*. Thus, in addition to *N *and *K*, our model requires 2*K *additional parameters, which we denote using the vectors *μ *and *π*. The *K *elements of the vector *π *are constrained to be greater than zero and sum to one. The *K *elements of *μ *are constrained to be greater than zero, monotonically increasing with increasing *k*, and that *μ_K _*has a fixed value of one (for the core genes in the supragenome). Our observed sample data from the *|S| *genomes under study (17 here) is represented by a vector C = {c_0_, c_1_, c_2_, ..., c_|S|_} whose elements equal the number of genes observed in exactly *n *= 0, 1, 2, ..., *|S| *of these genomes. A constrained nonlinear programming function (*fmincon*) from MatLab's Optimization Toolbox is used to maximize the following log-likelihood function of the observed gene frequencies using values of *N *over a reasonable range [20]:

Our initial model only carried out the optimization with respect to *N *and *π*; but did not allow the values of *μ_k _*(where *k *<*K*) to vary during the maximization of the log-likelihood of the observed data. The revised model removes this restriction, and the results obtained using this revised model yield insights not previously available.

**Figure 3:**
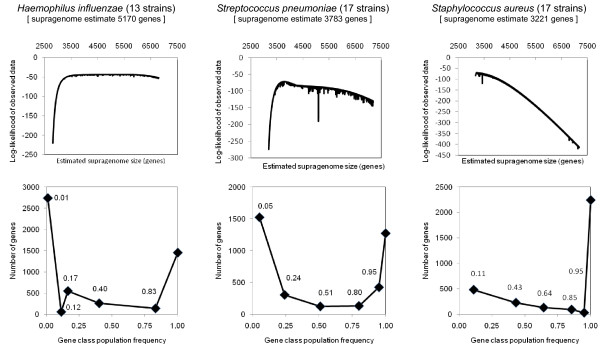
**Finite supragenome model results using (*K *= 6) variable population gene frequency classes**. In our previous supragenome analyses carried out with *Haemophilus influenzae *and *Streptococcus pneumoniae *we used a version of the finite supragenome model that required fixed population gene frequency classes. This model has been updated to make the optimization function (the log-likelihood of the observed sample gene frequency histogram, i.e., the observed gene frequency class distribution among the |*S*| strains examined) dependent on the values of the population gene frequency vector (*μ*) as well as the values of the corresponding mixture coefficient vector (*π*, for the probability that a gene in a supragenome will be represented in one of the *K *classes of population gene frequencies). For a given species, the bottom graph plots the values of the vector *μ *against the product of the estimate of supragenome size and the values of the vector *π*, all obtained at the maximization of the log-likelihood function.

An overview of the results obtained using the revised model is shown for three human bacterial pathogens: *Haemophilus influenzae, Streptococcus pneumoniae*, and *Staphylococcus aureus *(Figure 3). The results obtained for these three supragenomes differ significantly in their plots of the log-likelihood of the observed data against the values of supragenome size *N *that were examined during the optimization. Fortuitously, these results illustrate two contrasting types of supragenomes (*H. influenzae *and *S. aureus*) and a third (*S. pneumoniae*) whose general characteristics are intermediate between these two types. Thus, a broad plateau was observed in this plot for *H. influenzae*, whereas the log-likelihood plot for *S. aureus *declined very abruptly at estimated values of *N *that were significantly greater than the estimated size of its supragenome (Figure 3, upper panels). The revised supragenome model employed herein has the advantage that values of *μ_k _*(where *k *<*K*) are allowed to vary during the maximization of the log-likelihood. Hence *a priori *estimates of fixed values for these parameters (i.e., as was required in our initial supragenome model)--a procedure that the bottom panels of Figure 3 show is difficult--are conveniently avoided. At the extreme case of the lowest population gene frequency class, the values of *μ_1 _*and *π_1 _*at the maximization of the log-likelihood of the observed data indicate that the *H. influenzae *supragenome is dominated by a large pool of very rare genes. In contrast, the value for *μ_1 _*at the maximization of the log-likelihood of the observed data for the *S. aureus *supragenome (0.11) is an order of magnitude greater than that of *H. influenzae*. At the other extreme of population gene frequencies, even though the estimated size of the *S. aureus *supragenome at 3,221 chromosomal genes is the smallest value for *N *observed among these three species, the absolute number of *S. aureus *core genes (2,245) and their fraction of *N *(i.e., the value of *π_K _*= 0.6971) are both significantly greater than the same values for either *H. influenzae *or *S. pneumoniae *(Figure 3, lower panels). This estimate that approximately 30% of the *S. aureus *genes are non-core is in reasonable agreement with the results of earlier, more limited studies that used comparative genomic hybridization to estimate a value for this parameter of 22% [30].

**Figure 4:**
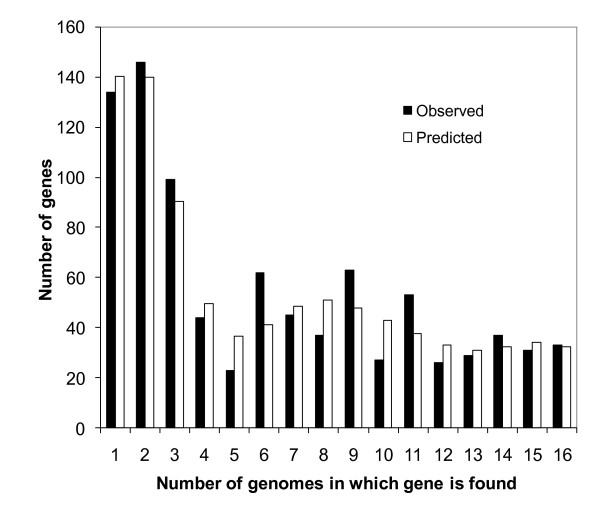
**Histogram of observed sample gene frequencies compared to the predicted number using the finite supragenome model**. The number of genes for each frequency class was calculated using the results from our revised finite supragenome model (trained on all 17 strains). The observed and predicted number of core genes (2,266) found in all 17 strains agreed exactly; these values are not shown to avoid distortion of the scale of the graph. Distributed genes appear in two or more strains, but not all (from 2 to 16 here).

**Figure 5:**
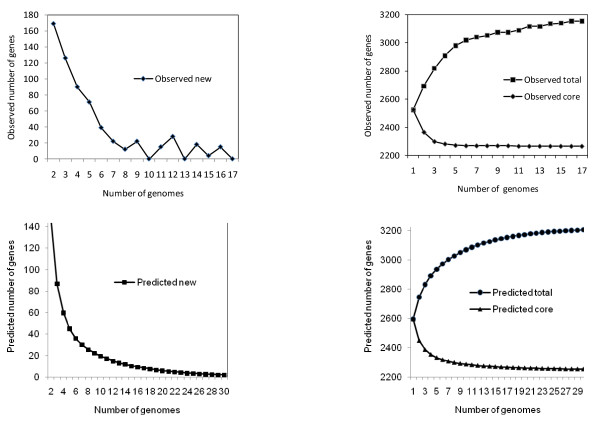
**Comparison of the observed and predicted supragenome parameters as additional strains are sequenced**. The two panels on the left show observed (upper panel) and predicted (lower panel) numbers of new genes that were or would be found in the second to the *n*th genome for the number of strains examined (17) or a hypothetical study of 30 strains of *Staphylococcus aureus*. The two panels on the right show observed (upper panel) and predicted (lower panel) numbers of core and total genes that were or are predicted to be found in second to the *n*th genome for the number of strains examined (17) or a hypothetical study of 30 strains of *Staphylococcus aureus*. Observed new, core, and total genes were calculated using genomes examined in ascending order of their counts of non-core genes.

The finite supragenome model is predictive as well as descriptive, Figure 4 shows the excellent correlation between the observed sample gene frequency data from the 17 *S. aureus *genomes under study (the number of genes observed in exactly *n *= 1, 2, ..., 17 of these genomes) and the same values predicted using the values of μ, π, and *N *obtained using our revised finite supragenome model trained on the sample data (all 17 strains). Figure 5 (lower panels) shows the ability of the model to predict the numbers of new, core, and the total number of chromosomal genes that should be detectable after sequencing up to 30 *S. aureus *genomes. These results agree very well with those obtained using the results from our analyses of the 17 *S. aureus *genomes under study (Figure 5, upper panels). They also indicate that the sequencing of 30 *S. aureus *genomes will yield 99.5% of the total number and 99.4% of the core chromosomal genes in this species' supragenome (*N *= 3,221 genes).

### Analyses of the RAST annotation data for the 17 *S. aureus *genomes under study

The RAST annotation data for the *S. aureus *chromosomal CDS agreed very well with the results of our CDS clustering procedure (Tables 4 and 5). Each CDS feature in a GenBank-style annotation record (such as the ones available from the RAST system) typically has a product feature qualifier value associated with it. Table 4 shows that 96% (3,038 of 3,155) of the orthologous clusters generated by our CGS supragenome analysis pipeline[21] were comprised of RAST-identified CDS that all mapped to a single product feature qualifier value. Similar results were obtained when confining the analyses to just the core clusters, i.e., the clusters with the largest numbers of members, where there was 98% (2,122 of 2,266) agreement between the two methods. In those cases where clusters were comprised of CDS that mapped to more than a single product feature qualifier value, the results were often understandable, e.g., the additional product qualifier values were either undefined (e.g., hypothetical protein) or imprecise (e.g., regulatory protein). The reverse mapping--of *S. aureus *RAST annotation product feature qualifiers to CGS-identified CDS clusters--was expected to be more problematic, but these results were also very robust (Table 5). In this analysis 82% (258 of 316) of the RAST annotation CDS product feature qualifiers that describe genes belonging to distributed clusters appeared in only a single cluster, and 91% (1,473 of 1,623) of the core cluster-derived CDS product feature qualifiers were found in only a single cluster. The difference between the results in Tables 5 and 4 indicate that the reverse mapping (of CDS product feature qualifier values to gene clusters) is slightly more susceptible to the problems cited above concerning imprecise CDS product descriptions.

**Table 4:** Mapping of *S. aureus *chromosomal CDS clusters to RAST annotation "Product" feature qualifiers

**Distinct product count**	Number of distinct Orthologous Clusters*			
	
	**Core**	**Distributed**	**Unique**	**Totals**
	
1	2,212	692	134	3038
2	41	56	0	97
3	11	6	0	17
> 3	2	1	0	3
Total	2,266	755	134	3155

*Shown are the numbers of orthologous clusters of core, distributed, or unique type whose constituent CDS yield the indicated numbers of distinct RAST annotation CDS "Product" feature qualifiers.

**Table 5:** Mapping of *S. aureus *RAST annotation "Product" feature qualifiers to chromosomal CDS clusters

**Distinct cluster count**	Number of distinct RAST "Product" Feature Qualifiers*			
	
	**Core**	**Distributed**	**Unique**	**Totals**
	
1	1,473	258	62	1793
2	122	39	7	168
3	17	10	2	29
> 3	11	9	1	21
Total	1,623	316	72	2011

*Shown are the numbers of distinct RAST annotation CDS "product" feature qualifiers that describe CDS belonging to either core, distributed, or unique clusters and where the relevant CDS yield the indicated number of distinct clusters. The CDS product feature qualifier "hypothetical protein" was deliberately excluded as it would be expected to map to different clusters.

Seven percent (220 of 3,155) of the chromosomal CDS clusters (genes) found in the 17 *S. aureus *genomes under study were comprised of CDS that were annotated as being phage-derived, and these genes were unevenly distributed among the core, distributed and unique gene sets. Thus, a query of the CDS product feature qualifier values for the term *phage *anywhere in the product name yielded a mapping of the CDS annotations to 7 core, 190 distributed, and 23 unique clusters. Thus, although 72% (2,266), 24% (755), and 4% (134) of the chromosomal CDS clusters found in the 17 *S. aureus *genomes under study represented core, distributed, and unique genes, 3%, 86%, and 11% of the phage-derived genes, respectively, were from these three classes of gene clusters.

The distinctive strategy of the RAST system is to have domain experts maintain well-curated *subsystem *functional annotations (e.g., metabolic pathways, regulatory pathways, or cellular structures) that can be used across bacterial genomes, instead of having the functional annotation of individual bacterial genomes attempted one-by-one by non-experts in the various subsystems that these genomes may encode. Subsystems analysis of the *S. aureus *chromosomal CDS annotations in the context of our gene clustering results was quite revealing (Table 6) in that almost half of the core CDS clusters could not be assigned to any RAST subsystem, as well as 90% of the distributed cluster CDS and 94% of the unique cluster CDS. These results highlight our very limited understanding of the biology of this important bacterial pathogen, as well as the need for improvements in functional annotation to keep pace with the extraordinary productivity of DNA sequencing technology.

**Table 6:** Supragenome Coding Sequence (CDS) Gene Assignments to RAST Subsystems

Subsystem annotation*	CDS count
**Core genes**	
• none	20,117
• Ribosome LSU bacterial	558
• Teichoic and lipoteichoic acids biosynthesis	385
• Heme, hemin uptake and utilization systems in Gram Positives	358
• Glycerolipid and Glycerophospholipid Metabolism in Bacteria	357
• DNA-replication	357
• Conserved gene cluster associated with Met-tRNA formyltransferase	357
• Ribosome SSU bacterial	357
• Peptidoglycan Biosynthesis	340
• tRNA modification *E.coli*	339
• Adhesins in Staphylococcus	320
• DNA repair, bacterial	311
• Methionine Biosynthesis	307
• tRNA aminoacylation	289
• Embden-Meyerhof and Gluconeogenesis	255
• Bacterial Cell Division	255
• pyrimidine conversions	244
• Translation factors bacterial	242
• Other defined categories (206 additional RAST subsystems)	14,724
**Distributed genes**	
◦ none	5,161
◦ Staphylococcal pathogenicity islands SaPI	68
◦ ABC transporter oligopeptide (TC 3.A.1.5.1)	62
◦ ESAT-6 proteins secretion system in Firmicutes	60
◦ Methicillin resistance in Staphylococci	47
◦ Adhesins in Staphylococcus	39
◦ Restriction-Modification System	33
◦ Cobalt-zinc-cadmium resistance	31
◦ Potassium homeostasis	27
◦ Teichoic and lipoteichoic acids biosynthesis	22
◦ Aminoglycoside adenylyltransferases	17
◦ Sex pheromones in *Enterococcus faecalis *and other Firmicutes	16
◦ DNA repair, bacterial	16
◦ tRNA modification E.coli	16
◦ Nudix proteins (nucleoside triphosphate hydrolases)	15
◦ Fosfomycin resistance	14
◦ Tn552	14
◦ Glycerol and Glycerol-3-phosphate Uptake and Utilization	12
◦ Peptidoglycan Biosynthesis	12
◦ Other defined categories (15 additional RAST subsystems)	60
**Unique genes**	
❖ none	130
❖ Restriction-Modification System	3
❖ Streptothricin resistance	1
❖ Teichoic and lipoteichoic acids biosynthesis	1
❖ ABC transporter oligopeptide (TC 3.A.1.5.1)	1
❖ Formaldehyde assimilation: Ribulose monophosphate pathway	1
❖ Heme and Siroheme Biosynthesis	1

*50% of core, 90% of distributed, and 94% of unique CDS could not be assigned to any RAST subsystem.

The RAST annotation data also provided useful insights into the presence or absence of genetic determinants of methicillin resistance (Table 7) in the genomes of the 17 *S. aureus *strains under study. Thirteen gene product descriptions indicated involvement of the CDS expression product in resistance to the antibiotic of choice for *S. aureus *infections, methicillin. Ten of these 13 genes were core genes, with the remaining three genes *mecA, mecI*, and *mecR1 *distributed among more than one but not all genomes (Table 7). If present, all of these genes were single copy, with the exception of the *fmtB *gene, which was present in multiple copies in 7 of the 17 genomes under study. The presence of the *mecA *gene was consistent with the known methicillin-resistance status of the strains, and its absence in the untested strain CGSSa00 indicated that this strain should be methicillin-susceptible.

**Table 7:** Chromosomal Coding Sequence (CDS) annotations associated with Methicillin Resistance

Genome	Sensitivity	FemA	FemB	FemC	FemD	FmtA	FmtB	FmtC	HmrA	HmrB	LytH	MecA	MecI	MecR1
CGSSa00	untested	1	1	1	1	1	3	1	1	1	1	0	0	0
CGSSa01	untested	1	1	1	1	1	2	1	1	1	1	1	0	1
CGSSa03	untested	1	1	1	1	1	1	1	1	1	1	1	1	1
COL	MRSA	1	1	1	1	1	1	1	1	1	1	1	0	1
JH1	VISA	1	1	1	1	1	1	1	1	1	1	1	1	1
JH9	VISA	1	1	1	1	1	1	1	1	1	1	1	1	1
MRSA252	MRSA	1	1	1	1	1	2	1	1	1	1	1	1	1
MSSA476	MSSA	1	1	1	1	1	2	1	1	1	1	0	0	0
Mu3	hetero-VISA	1	1	1	1	1	1	1	1	1	1	1	1	1
Mu50	HA-MRVISA	1	1	1	1	1	1	1	1	1	1	1	1	1
MW2	CA-MRSA	1	1	1	1	1	2	1	1	1	1	1	0	1
N315	MRSA	1	1	1	1	1	1	1	1	1	1	1	1	1
NCTC8325	MRSA	1	1	1	1	1	1	1	1	1	1	0	0	0
Newman	MSSA	1	1	1	1	1	1	1	1	1	1	0	0	0
RF122 (ET3-1)	sensitive	1	1	1	1	1	1	1	1	1	1	0	0	0
USA300 (FPR3757)	CA-MRSA	1	1	1	1	1	2	1	1	1	1	1	0	1
USA300TCH15	CA-MRSA	1	1	1	1	1	2	1	1	1	1	1	0	1

**Abbreviations **(see also Table 1): FemA, essential for MR (glycine interpeptide bridge formation); FemB, involved in MR (glycine interpeptide bridge formation; FemC, involved in MR (glutamine synthetase repressor); FemD (phosphoglucosamine mutase EC 5.4.2.10) involved in MR; FmtA, involved in MR (affects cell wall cross-linking and amidation); FmtB, (Mrp) involved in MR and cell wall biosynthesis; FmtC, (MrpF) involved in MR (L-lysine modification of phosphatidylglycerol); HmrA, involved in MR (amidohydrolase of M40 family); HmrB, Acyl carrier protein involved in MR; LytH, involved in MR (N-acetylmuramoyl-L-alanine amidase, EC 3.5.1.28 domain); MecA, Penicillin-binding protein PBP2a, MR determinant, transpeptidase; MecI, MR repressor; MecR1, MR regulatory sensor-transducer.

## Discussion

The first model for supragenome (or pan-genome) analysis [19] was developed using genomic DNA sequence datasets from eight strains of the species *Streptococcus agalactiae*, also known as group B *Streptococcus *(GBS). This model was developed by fitting an exponential decay function to a plot of the average number of *core *genes observed with increasing numbers of genomes examined (where the average was taken for all possible permutations of the order of consideration of the genomes under study), and took the asymptote defined by such a plot as an estimate of the size of the GBS *core *genome. This model also fitted a second exponential decay function to a plot of the average number of *new *genes observed with increasing numbers of genomes examined (where the average is taken as before), and took the asymptote as an estimate of the number of *new *genes that would be observed with each new GBS genome sequenced. Finally, this model also estimated the *size *of the GBS pan-genome by deriving a third equation for its rate of growth. A recent review [31] proposed a revised version of this model that adopts a power law fit (Heaps' Law, from the field of information retrieval) in lieu of the earlier exponential fit of the observed data. In both the original and the power law models, a threshold parameter (α) is used to distinguish *open *and *closed *pan-genomes, where an open pan-genome (with α ≤ 1) is defined as one that will yield a non-zero number of new genes when each additional genome of the species is sequenced. More recently, with the advent of ever less expensive sequencing technologies, making it possible to sequence scores of independent strains, it could be argued that modeling of the supra/pan-genome is unnecessary since sequencing of additional strains can be continued until no significant number of novel genes are identified [32]

The probabilistic foundation of the model used in this work [21] offers a somewhat different perspective, but with the improvements described above that take into account multiple gene frequency classes allows for accurate supragenome modeling of populations/species for which it is not possible to obtain multiple independent clonal lineages (i.e. unculturable organisms) for sequence analysis. In these cases gene frequency were inferred from the different sequence coverage levels observed within the sequenced population. Since the vast majority of bacterial species are not culturable, but are now amenable to whole genome sequencing through single cell isolation and whole genome amplification techniques [33] our model can be used to estimate the percentage of the supragenome that has been obtained at intermediate coverage levels.

A recent comparison of the pan-genome model of Tettelin et al. with our finite supragenome model demonstrated that the two models make highly similar predictions when provided the same dataset [26], thus serving as a validation for both. However, both models share fundamental challenges in areas such as the selection of appropriate genomic DNA sequence datasets to use. For example, we chose not to include *S. aureus *plasmid DNA sequences, e.g., those associated with the published genomes that were used in our analysis (Table 1), nor the DNA sequences of the many *S. aureus *bacteriophage genomes that have been published [34]. However, our analysis included three draft genomes from *S. aureus *strains that we sequenced, and these unfinished genomes may contain plasmid-derived contigs. The results in Table 3 in fact suggest that the strain CGSSa01 may contain one or more *S. aureus *plasmids. Its genome contains the largest number of genes (2,648), and the 18 unique genes it contains are significantly greater than all but two of the other 17 strains. A comprehensive review of the *S. aureus *genome [35] provides a detailed description of some of the many plasmids and other mobile elements that it may contain. Decisions about the inclusion or exclusion of published plasmid and bacteriophage DNA sequences in a supragenome analysis can therefore lead to systematic error in estimates of counts of different classes of genes (e.g., core and unique genes).

Other issues arise during the selection of appropriate genomic DNA sequence datasets to use in a supragenome analysis. In this work we included genomes that are known to be very closely related, e.g., those of strains JH1 and JH9 [28], as well as one known outlier genome (RF122) of bovine origin [36]. These decisions can also be criticized as leading to systematic error in estimates of counts of different classes of genes. However, inclusion of a limited number of closely related and outlier strains also provide for useful internal controls for the results of the supragenome analysis. In some respects, and especially for a species such as *S. aureus*, bias in the selection of genomic DNA sequence datasets to use in a supragenome analysis is unavoidable. Given the intense interest in clinically relevant strains of *S. aureus*, one can reasonably expect that even with the ever increasing affordability and subsequent availability of bacterial genomic DNA sequence datasets, the *S. aureus *strains selected for sequencing will for the foreseeable future be dominated by epidemiologically important clinical strains, and that the much more quantitatively representative commensal *S. aureus *stains that could be isolated from human subjects will be under-represented in supragenome and other comparative genomic analyses.

The *S. aureus *core genome has been found to contain a heptameric DNA sequence (GAAGCGG) that is believed to protect it from uncontrolled rearrangements [37]. This conserved *crossover hotspot initiator *or *chi *site is not the only DNA sequence motif and associated nucleic acid information processing system with a putative influence on the structure and maintenance of the *S. aureus *supragenome. The *Sau1 *Type I restriction-modification (RM) system has at least two important influences in this regard [38]. First, it reduces the efficiency of conjugation between *S. aureus *and enterococci, the putative source of vancomycin resistance. This is believed to explain why very few vancomycin-resistant *S. aureus *strains have arisen, despite tremendous selective pressure acting on the bacterial flora of patients treated with this drug [39]. Second, the *Sau1 *RM system's multi-copy specificity gene, *sau1hsdS*, has many alleles with significant population frequencies, and these alleles correspond to the major *S. aureus *lineages. Five copies of this gene occur in the RF122 genome; three copies in the genomes of CGSSa01, MSSA476, USA300, and USA300TCH15, and two copies in the remaining genomes we examined (i.e., it appears to be a *multi-copy core gene *found in all genomes examined). The *Sau1 *RM system therefore not only controls horizontal gene flow into *S. aureus *from other species, but also within *S. aureus *lineages via the polymorphic RM specificity alleles of *sau1hsdS *loci [38].

Analysis of the supragenome of *S. aureus *isolates from antibiotic-naïve populations would be an interesting extension of this work. Paradoxically, antibiotic treatment increases *S. aureus *conjugation frequency [40]; induces *S. aureus *temperate phage to excise, replicate, and transfer pathogenicity islands [41]; and when used in combination therapies may unexpectedly increase the spread of resistance among *S. aureus *strains [42]. In some bacterial genomes the selection pressure exerted by antibiotic exposure may also have the unexpected effect of promoting multi-drug resistance due to *positive *epistasis amongst combinations of alleles of antibiotic resistance loci [43]. Thus, one might expect that *S. aureus *isolates from antibiotic-naïve populations would yield an estimate of supragenome size smaller than that reported here, and be comprised of an even larger percentage of core genes. The results of a supragenome analysis may therefore represent an aggregate of the results of environmental niche-specific supragenomes affected by extrinsic agents such as antibiotics that modulate horizontal gene transfer, as well as regulatory allele-specific supragenomes affected by intrinsic genetic phenomena (such as the *Sal1 *system) that also modulate horizontal gene transfer.

Another interesting extension of this work would be an analysis of the supragenome of the related species *Staphylococcus sciuri*, which is one of the most abundant staphylococcal species and a frequent epidermal commensal of animals [44]. The *mecA *gene found in methicillin-resistant *S. aureus *(MRSA) strains encodes the PBP2A penicillin-binding protein, whose affinity for β-lactam antibiotics acts as a sink that vitiates the efficacy of these drugs and protects native *S. aureus *PBPs during their function as bacterial cell wall synthetic enzymes [45]. Incorporation of the *mecA *gene into the *S. aureus *genome is an unusual event, and requires both a delivery entity called the staphylococcal chromosome cassette (SCC), and a suitable but rarely encountered *S. aureus *genetic background that can tolerate the presence and expression of the *mecA *gene [45]. *Staphylococcus sciuri*, although susceptible to β-lactam antibiotics, is believed to be the source of the precursor homolog of the *mecA *gene present in the limited number of MRSA strains that have emerged worldwide [45]. Although limited in number, MRSA strains have spread in an epidemic manner with devastating clinical consequences. The *S. sciuri *genome appears to be ubiquitously agreeable to the presence and expression of its *mecA *precursor homolog [44], and hence a study of the *S. sciuri *supragenome may yield insights into the genetic determinants whose homologs in, or horizontal acquisition by, a *S. aureus *genome may predispose to the acquisition of *mecA*.

## Conclusions

The supragenome of *S. aureus *offers a significant contrast to those of other human bacterial pathogens including *H. influenzae *and *S. pneumoniae *which share the nasopharynx as their primary site of colonization. The *S. aureus *supragenome (3,221 chromosomal genes) was the smallest among these three species, yet it contained the largest number (2,245) of core genes (Figure 3). *H. influenzae *and *S. pneumoniae *each has a larger horizontal gene pool [45] to draw from by virtue of their possession of autocompetence and autotransformation systems which facilitate the horizontal acquisition of new genes [21,22]. Although some strains of *S. aureus *are deadly pathogens, most are not, and when compared to *H. influenzae *and *S. pneumoniae*, the *S. aureus *supragenome may have co-evolved with its human host for a longer period of time. As a result, there may be relatively less selection pressure on the *S. aureus *supragenome to maintain a larger size, as over an extended period of evolutionary time it has optimized its ability to maintain core and distributed phenotypes to survive the environmental conditions it typically encounters as a predominantly commensal species of its human host.

Finally, we are currently extending the bioinformatic analyses described herein with the development of a free, post-annotation software package for the execution of the full supragenome analysis pipeline described here. This project will provide the community with the ability to reproduce a given set of published supragenome analysis results, re-analyze the underlying data after modification of the parameters used during an analysis, and perform more detailed and insightful querying of the results than can be summarized in a typical journal publication.

## Methods

### DNA sequencing and genome assembly

The genomes of three *S. aureus *strains CGSSa00, CGSSa01, and CGSSa03 were sequenced at the Center for Genomic Sciences (CGS). All three are low-passage clinical isolates obtained from patients in Pittsburgh, and were obtained, respectively, from: (i) a nasal specimen from an asymptomatic individual; (ii) an elbow arthroplasty infection [46]; and (iii) a chronic abdominal mesh implant infection that developed after ventral herniorrhaphy [47]. Each high-coverage (~20X) draft genome assembly was obtained using data generated on a Roche/454 Life Sciences GS-FLX instrument using one region of a two-region 70 × 75 mm pico-titer plate [47]. *De novo *draft genome assemblies were obtained using the Newbler assembler, software releases 1.1.01.20 (CGSSa00) and 1.1.03.24 (CGSSa01 and CGSSa03). Newbler's default assembly parameters were used except for the *minimum overlap identity *(MOI) percentages (default = 90%) used for the CGSSa01 and CGSSa03 assemblies, which were 96% and 97%, respectively. For each genome the optimal Newbler genome assembly was chosen (from a series of assemblies using different MOI values) as the one that yielded the smallest number of large contigs, the longest overall assembly length, and the smallest percentage of *Q39minus *Newbler-estimated assembly base-call quality values. An initial round of gap closure of the genome assembly of strain CGSSa00 was carried out as described [22]. Table 1 provides information about these three genomes and the 14 previously sequenced *S. aureus *genomes used in the comparative and supragenome analyses. Genomic DNA sequence assembly accession numbers for all genomes (Table 2) and publicly available FASTA files for the 14 reference genomes used were obtained from the NCBI.

### Automated bacterial genome annotation and generation and identification of protein-encoding gene clusters

The RAST system (Rapid Annotation using Subsystem Technology; http://rast.nmpdr.org/) [24] using default parameters was used to provide a consistent set of automated genome annotations for the bacterial chromosome assemblies of all 17 strains. Annotation output datasets were downloaded, and then in-house developed software was used to parse the protein-encoding gene features and prepare tranches of FASTA-formatted input files (proteins, genes, and genomes) for all-against-all sequence alignment jobs using the FASTA and TFASTY software [27] installed on the Codon computing cluster at the Pittsburgh Supercomputing Center, all as described [21,22]. Subsequent steps in the protein-encoding gene-clustering generation and identification procedures were also performed as described [21,22], with the exception that version 2 (instead of version 1) of the multiple sequence alignment program Partial Order Alignment (POA2) [27] was used during the CDS gene clustering procedure.

### Neighbor Grouping of *S. aureus *genomes

After the core (orthologs present in all genomes), distributed (orthologs present in two or more but not all genomes), and unique (present in only one genome) genes were identified, the presence or absence of the distributed genes in each of the 17 strains was used to define neighbor groups of the genomes under study, as described [23]. Briefly, *neighbor grouping *examines a pair-wise distance matrix in which the distance between a given pair of genomes is equal to the fraction of the distributed genes that is either present in both genomes or absent in both genomes. A pair of genomes are *neighbors *if the distance between them is less than the mean distance between each pair of genomes under study, minus the standard error of the mean. A valid *neighbor group *is a sub-graph comprised of two or more nodes (genomes) that are connected by *nearest-neighbor *edges.

### Mathematical modeling of the S. aureus supragenome

MatLab (version 7.1) and its Optimization Toolbox were used to develop a revised finite supragenome model [21] that allows the estimates of the population gene frequencies (for a limited number *K *of classes of genes) to vary during the maximization of the log-likelihood of the observed sample gene frequency data. The number of elements (*K*) of the population gene frequency vector (*μ*) and the corresponding mixture coefficient vector (*π*) was set at 6; the initial values of *π *were all set to 1/*K*, and the initial values of *μ *were {0.1, 0.3, 0.5, 0.7, 0.9, 1.0}. The MatLab programs used for the revised finite supragenome model will be provided upon request.

## Competing interests

The authors declare that they have no competing interests.

## Authors' contributions

RB performed the bioinformatic analyses and wrote the paper; AA performed the genome sequencing and genome assembly; BJ performed genome sequencing and annotations; JE wrote bioinformatic analysis programs and performed analyses; BGH wrote bioinformatic analysis programs; JH wrote developed the original and modified finite supragenome model; NLH performed bioinformatic analyses; EP designed and carried out gap closure and genome finishing studies; JH performed the microbiology and molecular biology experiments; SY wrote bioinformatic analysis programs; SK provided clinical strains of *S. aureus*; PS characterized the *S. aureus *stains; JCP edited the manuscript and provided funding for the study; GDE organized the study, analyzed the data, wrote the paper and provided funding; FZH conceived the study and oversaw the strain selection, genome sequencing and gap closure, writing of the bioinformatic analysis programs, and provided overall co-ordination of the project.

## References

[B1] CDC (2002) Staphylococcus aureus resistant to vancomycin--United States, 2002 MMWR 2002 51 565 567 12139181

[B2] Kluytmans J van Belkum A Verbrugh H Nasal carriage of Staphylococcus aureus: epidemiology, underlying mechanisms, and associated risks Clin Microbial Rev 1997 10 505 520 10.1128/cmr.10.3.505 PMC172932 9227864

[B3] Coates T Bax R Coates A Nasal decolonization of Staphylococcus aureus with mupirocin: strengths, weaknesses and future prospects J Antimicrob Chemother 2009 64 9 15 10.1093/jac/dkp159 19451132 PMC2692503

[B4] Daum RS Clinical practice. Skin and soft-tissue infections caused by methicillin-resistant Staphylococcus aureus N Engl J Med 2007 357 380 390 10.1056/NEJMcp070747 17652653

[B5] Stryjewski ME Chambers HF Skin and soft-tissue infections caused by community-acquired methicillin-resistant Staphylococcus aureus Clin Infect Dis 2008 46 Suppl 5 S368 377 18462092 10.1086/533593

[B6] Otto M Targeted immunotherapy for staphylococcal infections: focus on anti-MSCRAMM antibodies BioDrugs 2008 22 27 36 10.2165/00063030-200822010-00003 18215088

[B7] Klevens RM Morrison MA Nadle J Petit S Gershman K Ray S Harrison LH Lynfield R Dumyati G Townes JM Craig AS Zell ER Foshein GE McDougal LK Carey RB Fridkin SK Active Bacterial Core surveillance (ABCs) MRSA Investigators Invasive methicillin-resistant Staphylococcus aureus infections in the United States JAMA 2007 298 1763 1771 10.1001/jama.298.15.1763 17940231

[B8] Bancroft EA Antimicrobial resistance: it's not just for hospitals JAMA 2007 298 1803 1804 10.1001/jama.298.15.1803 17940239

[B9] CDC (2007) HIV/AIDS Surveillance Report Rev ed. Atlanta 2005 17

[B10] CDC (2007) Invasive methicillin-resistant Staphylococcus aureus infections among dialysis patients--United States MMWR 2005 56 197 199 17347644

[B11] Leonard FC Markey BK Meticillin-resistant Staphylococcus aureus in animals: a review Vet J 2008 175 27 36 10.1016/j.tvjl.2006.11.008 17215151

[B12] Nemati M Hermans K Lipinska U Denis O Deplano A Struelens M Devriese LA Pasmans F Haesebrouch F Antimicrobial resistance of old and recent Staphylococcus aureus isolates from poultry: first detection of livestock-associated methicillin-resistant strain ST398 Antimicrob Agents Chemother 2008 52 3817 3819 10.1128/AAC.00613-08 18663024 PMC2565892

[B13] Ehrlich GD The Biofilm and Distributed Genome Paradigms Provide a New Theoretical Structure for Understanding Chronic Bacterial Infections41st Interscience Conference on Antimicrobial Agents and Chemotherapy. Chicago, IL 2001 2001 American Society for Microbiology 524

[B14] Ehrlich GD Hu FZ Shen K Stoodley P Post JC Bacterial plurality as a general mechanism driving persistence in chronic infections Clin Orthop Relat Res 2005 437 20 24 16056021 10.1097/00003086-200508000-00005 PMC1351326

[B15] Hu FZ Ehrlich GD Population-level virulence factors amongst pathogenic bacteria: relation to infection outcome Future Microbio 2008 3 31 42 10.2217/17460913.3.1.31 18230032

[B16] Shen K Antalis P Gladitz J Sayeed S Ahmed A Yu S Hayes J Johnson S Dice B Dopico R Keefe R Janto B Chong W Goodwin J Wadowsky RM Erdos G Post JC Ehrlich GD Hu FZ Identification, distribution, and expression of novel genes in 10 clinical isolates of nontypeable Haemophilus influenzae Infect Immun 2005 73 3479 3491 10.1128/IAI.73.6.3479-3491.2005 15908377 PMC1111819

[B17] Shen K Gladitz J Antalis P Dice B Janto B Keefe R Hayes J Ahmed A Dopico R Ehrlich N Jocz J Kropp L Yu S Nistico L Greenberg DP Barbadora K Preston RA Post JC Ehrlich GD Hu FZ Characterization, distribution, and expression of novel genes among eight clinical isolates of Streptococcus pneumoniae Infect Immun 2006 74 321 330 10.1128/IAI.74.1.321-330.2006 16368987 PMC1346598

[B18] Shen K Sayeed S Antalis P Gladitz J Ahmed A Dice B Janto B Dopico R Keefe R hayes J Johnson S Yu S Ehrlich N Jocz J Kropp L Wong R Wadowsky RM Slifkin M Preston RA Erdos G Post JC Ehrlich GD Hu FZ Extensive genomic plasticity in Pseudomonas aeruginosa revealed by identification and distribution studies of novel genes among clinical isolates Infect Immun 2006 74 5272 5283 10.1128/IAI.00546-06 16926421 PMC1594838

[B19] Tettelin H Masignani V Cieslewicz MJ Donati C Medini D Ward NL Angiouli SV Crabtree J Jones AL Durkin AS Deboy RT Davidsen TM Mora M Scarselli M Margarit Ros yI Peterson JD Hauser CR Sundaram JP Nelson WC Madupu R Brinkac LM Dodson RJ Rosowitz MJ Sullivan SA Daugherty SC Haft DH Selengut J Gwinn ML Zhou L Zafar N Genome analysis of multiple pathogenic isolates of Streptococcus agalactiae: implications for the microbial "pan-genome" Proc Natl Acad Sci USA 2005 102 13950 13955 10.1073/pnas.0506758102 16172379 PMC1216834

[B20] Ehrlich GD Ahmed A Earl J Hiller NL Costerton JW Stoodley P Post JC DeMeo P Hu FZ The Distributed Genome Hypothesis as a Rubric for Understanding Evolution *in situ *During Chronic Infectious Processes FEMS Immunol Med Microbiol 2010 59 269 279 20618850 10.1111/j.1574-695X.2010.00704.x PMC2910629

[B21] Hogg JS Hu FZ Janto B Boissy R Hayes J Keefe R Post JC Ehrlich GD Characterization and modeling of the Haemophilus influenzae core and supragenomes based on the complete genomic sequences of Rd and 12 clinical nontypeable strains Genome Biol 2007 8 R103 10.1186/gb-2007-8-6-r103 17550610 PMC2394751

[B22] Hiller NL Janto B Hogg JS Boissy R Yu S Powell E Keefe R Ehrlich NE Shen K Hayes J Barbadora K Klimke W Dernovoy D Tatusova T Parkhill J Bentley SD Post JC Ehrlich GD Hu FZ Comparative genomic analyses of seventeen Streptococcus pneumoniae strains: insights into the pneumococcal supragenome J Bacteriol 2007 189 8186 81895 10.1128/JB.00690-07 17675389 PMC2168654

[B23] Hall BG Ehrlich GD Hu FZ Pan-genome analysis provides much higher strain typing resolution than does MLST Microbiology 2010 156 1060 1068 10.1099/mic.0.035188-0 20019077 PMC2889442

[B24] Aziz RK Bartels D Best AA DeJongh M Disz T Edwards RA Formsma K Gerdes S Glass EM Kubal M Meyer F Olsen GJ Olson R Osterman AL Overbeek RA McNeil LK Paarmann D Paczian T Parrello B Pusch GD Reich C Stevens R Vassieva O Vonstein V Wilke A Zagnitko O The RAST Server: rapid annotations using subsystems technology BMC Genomics 2008 9 75 10.1186/1471-2164-9-75 18261238 PMC2265698

[B25] Margulies M Egholm M Altman WE Attiya S Bader JS Bemben LA Berka J Braverman MS Chen YJ Chen Z Dewell SB Du L Fierro JM Gomes XV Godwin BC He W Helgesen S Ho CH Irzyk GP Jando SC Alenquer ML Jarvie TP Jirage KB Kim JB Knight JR Lanza JR Leamon JH Lefkowitz SM Lei M Li J Genome sequencing in microfabricated high-density picolitre reactors Nature 2005 437 376 380 16056220 10.1038/nature03959 PMC1464427

[B26] Donati C Hiller NL Tettelin H Muzzi A Croucher NJ Angiuoli SV Oggioni M Riley D Covacci A Bentley SD Kilian M Ehrlich GD Hu FZ Rappuoli R Moxon ER Masignani V Structure and dynamics of the pan-genome of Streptococcus pneumoniae and closely related species Genome Biol 2010 11 R107 10.1186/gb-2010-11-10-r107 21034474 PMC3218663

[B27] Grasso C Lee C Combining partial order alignment and progressive multiple sequence alignment increases alignment speed and scalability to very large alignment problems Bioinformatics 2004 20 1546 1556 10.1093/bioinformatics/bth126 14962922

[B28] Mwangi MM Wu SW Zhou Y Sieradzki K de Lencastre H Richardson P Bruce D Rubin E Myers E Siggia ED Tomasz A Tracking the in vivo evolution of multidrug resistance in Staphylococcus aureus by whole-genome sequencing Proc Natl Acad Sci USA 2007 104 9451 9456 10.1073/pnas.0609839104 17517606 PMC1890515

[B29] Baba T Takeuchi F Kuroda M Yuzawa H Aoki K Oguchi A Nagai Y Iwama N Asano K Naimi T Kuroda H Cui L Yamamoto K Hiramatsu K Genome and virulence determinants of high virulence community-acquired MRSA Lancet 2002 359 1819 1827 10.1016/S0140-6736(02)08713-5 12044378

[B30] Fitzgerald JR Sturdevant DE Mackie SM Gill SR Musser JM Evolutionary genomics of Staphylococcus aureus: insights into the origin of methicillin-resistant strains and the toxic shock syndrome epidemic Proc Natal Acad Sci USA 2001 98 8821 8826 10.1073/pnas.161098098 PMC37519 11447287

[B31] Tettelin H Riley D Cattuto C Medini D Comparative genomics: the bacterial pan-genome Current Opin Microbiol 2008 11 472 477 10.1016/j.mib.2008.09.006 19086349

[B32] Lefebure T Pavinski Bitar PD Suzuki H Stanhope MJ Evolutionary dynamics of complete Campylobacter Pan-Genomes and the bacterial species concept Genome Biology and Evolution 2010 2 646 655 10.1093/gbe/evq048 20688752 PMC2940326

[B33] Mussmann M Hu FZ Richter M de Beer D Preisler A Jørgensen BB Huntemann M Glöckner FO Amann R Koopman WJ Lasken RS Janto B Hogg J Stoodley P Boissy R Ehrlich GD Insights into the genome of large sulfur bacteria revealed by analysis of single filaments PLoS Biol 2007 9 e230 10.1371/journal.pbio.0050230 PMC1951784 17760503

[B34] Kwan T Liu J DuBow M Gros P Pelletier J The complete genomes and proteomes of 27 Staphylococcus aureus bacteriophages Proc Natl Acad Sci USA 2005 102 5174 5179 10.1073/pnas.0501140102 15788529 PMC556006

[B35] Młynarczyk A Młynarczyk G Jeljaszewicz J The genome of Staphylococcus aureus: a review Zentralb Bakteriol 1998 287 277 314 10.1016/s0934-8840(98)80165-5 9638861

[B36] Herron-Olson L Fitzgerald JR Musser JM Kapur V Molecular correlates of host specialization in Staphylococcus aureus PloS One 2007 2 e1120 10.1371/journal.pone.0001120 17971880 PMC2040198

[B37] Halpern D Chiapello H Schbath S Robin S Hennequet-Antier C Gruss A El Karoui M Identification of DNA motifs implicated in maintenance of bacterial core genomes by predictive modeling PLoS Genet 2007 3 1614 1621 17941709 10.1371/journal.pgen.0030153 PMC1976330

[B38] Waldron DE Lindsay JA Sau1: a novel lineage-specific type I restriction-modification system that blocks horizontal gene transfer into Staphylococcus aureus and between S. aureus isolates of different lineages J Bacteriol 2006 188 5578 5585 10.1128/JB.00418-06 16855248 PMC1540015

[B39] Weigel LM Clewell DB Gill SR Clark NC McDougal LK Flannagan SE Kolonay JF Shetty J Killgore GE Tenover FC Genetic analysis of a high-level vancomycin-resistant isolate of Staphylococcus aureus Science 2003 302 1569 1571 10.1126/science.1090956 14645850

[B40] Evans J Dyke KG Characterization of the conjugation system associated with the Staphylococcus aureus plasmid pJE1 J Gen Microbiol 1988 134 1 8 2846752 10.1099/00221287-134-1-1

[B41] Ubeda C Maiques E Knecht E Lasa I Novick RP Penadés JR Antibiotic-induced SOS response promotes horizontal dissemination of pathogenicity island-encoded virulence factors in staphylococci Mol Microbiol 2005 56 836 844 10.1111/j.1365-2958.2005.04584.x 15819636

[B42] Michel J Yeh PJ Chait R Moellering RC Kishony R Drug interactions modulate the potential for evolution of resistance Proc Natl Acad Sci USA 2008 105 14918 14923 10.1073/pnas.0800944105 18815368 PMC2567468

[B43] Trindade S Sousa A Xavier KB Dionisio F Ferreira MG Gordo I Positive epistasis drives the acquisition of multidrug resistance PLoS Genet 2009 5 e1000578 10.1371/journal.pgen.1000578 19629166 PMC2706973

[B44] Couto I de Lencastre H Severina E Kloos W Webster JA Ubiquitous presence of a mecA homologue in natural isolates of Staphylococcus sciuri Microbial drug resistance (Larchmont, N.Y.) 1996 2 377 91 10.1089/mdr.1996.2.377 9158808

[B45] de Lencastre H Oliveira D Tomasz A Antibiotic resistant Staphylococcus aureus: a paradigm of adaptive power Curr Opin Microbiol 2007 10 428 435 10.1016/j.mib.2007.08.003 17921044 PMC2701899

[B46] Slater FR Bailey MJ Tett AJ Turner SL Progress towards understanding the fate of plasmids in bacterial communities FEMS Microbiol Ecol 2008 66 3 13 10.1111/j.1574-6941.2008.00505.x 18507680

[B47] Stoodley P Nistico L Johnson S Lasko L Baratz M Gahlot V Ehrlich GD Kathju S Direct demonstration of viable Staphylococcus aureus biofilms in an infected total joint arthroplasty. A case report J Bone Joint Surg Am 2008 90 1751 1758 10.2106/JBJS.G.00838 18676908 PMC2729478

[B48] Pearson WR Flexible sequence similarity searching with the FASTA3 program package Methods Mol Biol 2000 132 185 219 10547837 10.1385/1-59259-192-2:185

